# Intestinal Elastography in the Diagnostics of Ulcerative Colitis: A Narrative Review

**DOI:** 10.3390/diagnostics12092070

**Published:** 2022-08-26

**Authors:** Maciej Cebula, Jakub Kufel, Anna Grażyńska, Justyna Habas, Katarzyna Gruszczyńska

**Affiliations:** 1 Department of Radiology and Nuclear Medicine, Faculty of Medical Sciences in Katowice, Medical University of Silesia, 40-754 Katowice, Poland; 2 Department of Biophysics, Faculty of Medical Sciences in Zabrze, Medical University of Silesia, 40-752 Katowice, Poland; 3 Department of Imaging Diagnostics and Interventional Radiology, Prof. Kornel Gibiński Independent Public Central Clinical Hospital, Medical University of Silesia in Katowice, 40-514 Katowice, Poland; 4 Faculty of Pharmaceutical Sciences in Sosnowiec, Medical University of Silesia, 40-055 Katowice, Poland

**Keywords:** ulcerative colitis, ultrasound elastography, transabdominal elastography, elastography

## Abstract

Ulcerative colitis (UC) is an inflammatory bowel disease (IBD) that mainly affects developed countries, but the number of cases in developing countries is increasing. We conducted a narrative review on the potential application of ultrasound elastography in the diagnosis and monitoring of UC, as this newly emerging method has promising results in other gut diseases. This review fulfilled the PRISMA Statement criteria with a time cut-off of June 2022. At the end of the review, of the 1334 identified studies, only five fulfilled all the inclusion criteria. Due to the small number of studies in this field, a reliable assessment of the usefulness of ultrasound elastography is difficult. We can only conclude that the transabdominal elastography examination did not significantly differ from the standard gastrointestinal ultrasonography examination and that measurements of the frontal intestinal wall should be made in the longitudinal section. The reports suggest that it is impossible to estimate the clinical scales used in disease assessment solely on the basis of elastographic measurements. Due to the different inclusion criteria, measurement methodologies, and elastographic techniques used in the analysed studies, a reliable comparative evaluation was impossible. Further work is required to assess the validity of expanding gastrointestinal ultrasonography with elastography in the diagnosis and monitoring of UC.

## 1. Introduction

Ulcerative colitis (UC) is an idiopathic inflammatory bowel disease (IBD) that affects the colon and rectum. This long-term condition is most common in industrialised nations, but lately, new cases are frequently occurring all over the world [1]. In the 20th Century, IBD, including UC, was mainly a disease affecting the rich countries of North America, Europe, and Oceania. In the 21st Century, the prevalence of this disease continues to increase in these regions, but it has also started to escalate in the heavily industrialised countries of Asia, Africa, and South America. It is estimated that the prevalence of UC is expected to intensify to become a major global problem and a great challenge for health systems around the world [2]. It has a characteristic age distribution of disease onset with two peaks, first at 20–30 years of age and second at 50–80 years of age [3]. The risk factors that have been reported so far are family history, specific ethnicity, highly processed diet, and intestinal dysbiosis [4,5]. However, the protective effect of appendectomy has been proven [6]. UC is often associated with other gastrointestinal conditions, such as colorectal cancer. Therefore, a screening colonoscopy is recommended [7]. The lifetime healthcare costs incurred by patients with UC are estimated to be USD 405,496, with the highest lifetime cost burden when diagnosed under 11 years of age [8].

Due to the high disease incidence and risk to the health and life of patients, imaging methods are used to evaluate potential complications. With the role of established, attainable methods, such as plain abdominal radiography, double-contrast barium enema, abdominal computed tomography, and magnetic resonance imaging, the role of the remaining, especially new, imaging methods remains unexplored [9,10,11]. One such method is ultrasound elastography, which has been introduced in recent years.

Elastography is a non-invasive method that uses ultrasound to assess tissue stiffness. There are two main types of elastography: shear wave elastography (SWE) and real-time elastography (RTE), also known as strain elastography (SE). SWE is based on an acoustic radiation force impulse (ARFI) that propagates through tissue and subsequently assesses its elastic properties by measuring the velocity of the shear wave. This method enables a reproducible, objective, and quantitative evaluation of tissue stiffness. The assessment of tissues using SE involves a comparison between the targeted and surrounding tissues after external pressure has been induced by an ultrasound operator. The semi-quantitative results of SE are presented as a colour-coded elastogram, a map illustrating elastic strains with colour gradation [12,13,14]. The stiffness thresholds for specific equipment should not be utilised for other equipment [15]. The preparation for elastographic examination does not differ from that before intestinal ultrasound; thus, the assessment may be carried out simultaneously. Thus far, the profitability of ultrasound elastography has been proven in relation to liver examinations [16]. To the best of our knowledge, there are no studies on this aspect in relation to intestinal elastography. It is worth noting that elastography is covered by the as low as reasonably achievable (ALARA) principle because of the relatively high energy required for tissue displacement, energy deposition, and possible temperature rise. [17].

Due to promising reports on the use of ultrasound in the diagnostics of other diseases, we examined whether the additional use of ultrasound elastography could provide some benefits in the diagnostics or monitoring process of UC [18,19,20] and conducted a narrative review.

## 2. Methods

The narrative review was conducted in accordance with the latest PRISMA 2020 statement guidelines [21]. The checklist from this review is an indispensable part of the study presented in Supplement S1.

### 2.1. Search Strategy and Selection Criteria

**Figure 1:**
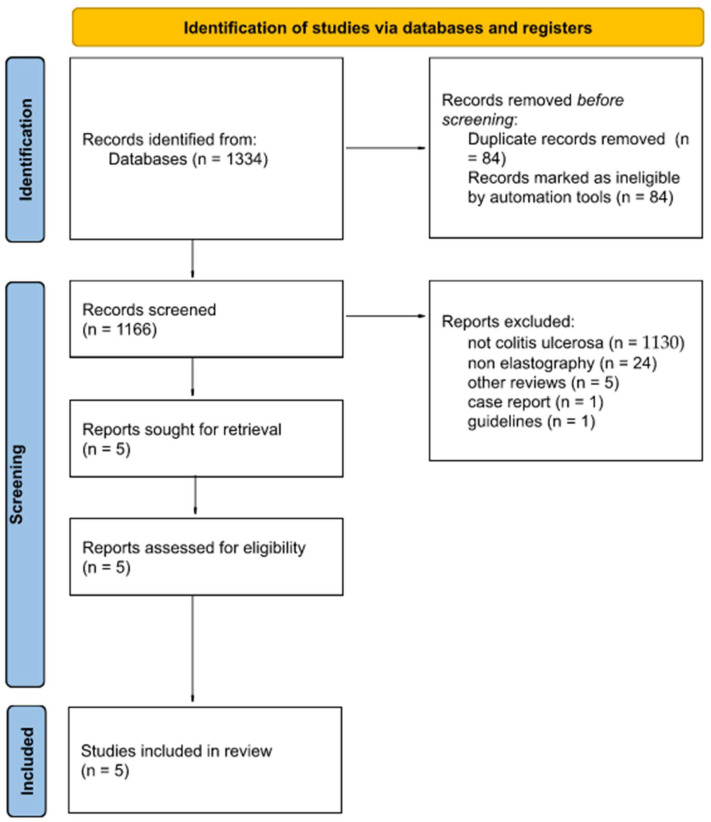
Flow diagram showing the course of the narrative review, taking into account the number of articles at a given stage of work.

For this study, the following databases were searched until June 2022: MED-LINE via PubMed, Embase, Scopus, and Cochrane. The search strategy was developed according to repeatable patterns, ensuring the high quality of the obtained results. The database search strategy is presented in Figure 1. The search was carried out using the following medical subject heading keywords that ensured the repeatability of the search in various searched databases: ultrasonic transverse wave, elastography, elastograms, elastographs, and ulcerative colitis.

The search was performed using various filters: >2010, open access, English, and human studies. In the Web of Science database, an additional filter was used to refine the results: radiology nuclear medicine medical imaging. However, in the Scopus database, only medicine and article were used. Only published original articles were included in the review, and preprints and other reviews were not included. The entire search strategy, including the individual phrases entered in the medical database search engines and the exact number of articles during the search and after using filters, is presented in Supplement S2.

The flow diagram in Figure 1 shows the number of articles included and excluded in each stage of the narrative review, ensuring greater accuracy and reliability.

### 2.2. Data Extraction and Quality Assessment

Every article found was exported from the database and then imported to the Rayyan Qatar Computing Research Institute. This highly specialised, web-based software was designed to carry out narrative reviews using several judges and the possibility of blinding the works, which translates into the high credibility of the results of this work [22]. The articles were evaluated by two independent reviewers for suitability in this study. A total of 84 duplicate articles were excluded. Articles that were not relevant to colitis ulcerosa (1130) or elastography (24), other reviews (5), case reports (1), and guidelines (1) were also excluded from the study. All the conflicts resulting from misunderstandings between the two reviewers were eventually solved by a third independent reviewer after blinding their decision. In total, five studies fulfilled all the assumed criteria. Cohen’s kappa (κ) was estimated to be 0.43 (agreement in 99.3%), which is interpreted as a moderate agreement between the authors [23].

#### 2.2.1. Detailed Inclusion Criteria

The articles included in the study met all of the following conditions cumulatively: English language; access to the full version of the article; the article should have an abstract in English; and the article is focused on elastography and UC. In addition, the content of the article should be sufficient to enable a cross-article comparison for the purposes of the review.

#### 2.2.2. Detailed Exclusion Criteria

The articles that were not included in this study met at least one criterion: the content of the article was about a topic other than elastography; the article dealt with diseases other than UC; no abstract; paid articles (OA notes); no access to the full article; and non-English articles.

## 3. Results

Out of the five studies that qualified for the narrative review, three were prospective studies [24,25,26], one was a retrospective study [27], and one met the criteria for a retrospective and prospective study [28]. The articles were published between 2010 and 2022. A total of 238 patients participated in the research. In this group, 142 patients had UC, 46 had Crohn’s disease (CD), and 48 were healthy controls [24,25,26,27,28]. It was not possible to analyse the age of the patients because of differences in reporting methods. Goertz et al.’s [24] study did not provide information about the patients’ ages.

### 3.1. Aim of the Studies

Goertz et al. [24] performed elastographic measurements within the intestinal wall using the ARFI method in healthy volunteers and patients with UC. The ARFI shear wave velocities of the terminal ileum and the colonic frame segments were analysed and correlated with the findings of the B-mode sonography of the bowel wall. Rustemovic et al. [27] evaluated the role of transrectal elastography in distinguishing between CD and UC. Ishikawa et al. [28] investigated whether the differences in the tissue strain of the mucosal layer obtained using elastography appropriately reflected the colonoscopic findings and correlated with disease activity among UC patients. Yamada et al. [25] examined the relationship between the activities of UC and SWE and shear wave dispersion (SWD). Marin et al. [26] analysed the clinical significance of IUS and SWE in monitoring inflammatory bowel diseases by developing an ultrasound score to predict disease activity in patients with CD and UC.

### 3.2. Activity of the Disease, Operations, and Additional Circumstances in Which the Studies Were Conducted

Goertz et al. [24] assessed the clinical stage of UC advancement using the clinical Mayo Subscore scale and found that patients had 6.6 ± 1.7 points on average and that the average stage of UC was moderate. Rustemovic et al. [27] evaluated patients using the Baron Score endoscopic scale and reported that 11 patients scored 0 point, 3 patients scored 1 point, 11 patients scored 2 points, and 0 patient scored 3 points. In the case of Ishikawa et al. [28], patients were clinically examined using the clinical activity index, and 16 patients were found to be in remission and 21 in active UC. Rustemovic et al. [27] investigated CD patients whose advancement of the disease was graded by an endoscopist based on the endoscopic appearance as “remission” if no visible lesions were seen, “mild activity” if the erythematous mucosa and/or erosions of the mucosa were seen, but with no ulcers in the rectum, and as “severe activity” if ulcerations or spontaneous bleeding was observed.

Yamada et al. [25] rated the stage of UC using the Lichtiger index, and the patients scored eight points on average (IQR 5.3–10.8). Additionally, the ulcerative colitis endoscopic index was used to evaluate endoscopic activity, and the patients scored four points on average (IQR 3.3–5). Conversely, in Marin et al.’s [26] study, disease activity was rated using inflammatory markers, such as C-reactive protein (approximately 3.55 µg/dL), faecal calprotectin (approximately 320 µg/dL), and the Mayo Score (average score of 5.5 points).

### 3.3. Experience and Number of Operators

In Goertz et al.’s [24] study, all elastographic examinations were performed by one radiologist who had more than six years of experience in performing elastography (DEGUM Qualification Level 2). In Rustemovic et al. [27], all studies were carried out by one radiologist, whose seniority and experience in intestinal elastography were not reported. In Ishikawa et al. [28], the examinations were performed by one physician specialising in elastography. In Yamada et al. [25], the examinations were conducted by one experienced gastroenterologist who performed 3000 ultrasound examinations and belonged to the Japan Society of Ultrasonics in Medicine. The gastroenterologist was blinded to the endoscopic findings and clinical symptoms, but not to the diagnosis of UC. In Marin et al. [26], all medical examinations were performed by a gastroenterologist who had more than five years of experience in performing elastography. 

### 3.4. Sonoelastography Technique, Region of Interest, and Parameters

#### 3.4.1. Investigated Area

Goertz et al. [24] targeted locations such as the terminal ileum and the ascending, transverse, descending, and sigmoid colon. Rustemovitz et al. [27] and Ishikawa [28] examined the rectum and the descending colon, respectively. Yamada et al. [25] made all the SWE/SWD measurements on the sigmoid. In Marin et al.’s [26] case, the entire length of the colon was examined. 

#### 3.4.2. Devices used in Examinations

Goertz et al. [24] used an Acuson S2000 ultrasound device (Siemens Medical Solution, software version VB21A, Erlangen, Germany) with a linear transducer 9L4 for analysis. The equipment in Rustemovic et al.’s [27] study was a linear echo-endoscope (Pentax FG-38 UX) with probes of 7.5−12 MHz (Hitachi EUB 8500). Ishikawa et al. [28] used a Hitachi EUB-8500 US system (Hitachi Medical, Tokyo, Japan), in which the object was observed using a linear probe (EUP-L52, 3.5Y7.5 MHz). For SWE and SWD measurements, Yamada et al. [25] used an Aplio i900 ultrasound machine (Canon Medical Systems Corp.) with a convex probe (i8CX1). Marin et al. [26] performed measurements using an Acuson S2000 (version VB20, model no. 10041461, Siemens Medical Solutions USA, Mountain View, CA, USA) with a 3–10 MHz linear transducer and a 4 C1 transducer (4 MHz) in the most affected bowel segment.

#### 3.4.3. Patients’ Preparation and Conduction of Examination

In Goertz et al.’s [24] study, during the examination by a physician, the patients were in a supine position with a relaxed breath-hold during measurements. The healthy volunteers fasted for > 8 h. Relevant bowel wall movements possibly affecting elastography were visually excluded. The contact pressure of the transducer was just high enough to visualise the designated locations. In Rustemovic et al. [27], each patient was examined in the left lateral decubitus position, without previous preparation. The probe was covered with a condom and inserted in the ampulla of the rectum under direct vision. A 170° linear probe display of the rectal wall and surrounding tissue was provided. In Ishikawa et al. [28], elastography was performed just before endoscopic examination and after the ingestion of an oral electrolyte lavage solution. Compressing the abdominal wall with the ultrasound probe also compressed the descending colon, and EG-mode images of the descending colon were clearly obtained. Total colonoscopy was performed after the completion of the elastography examination, with careful observation of the mucosa of the descending colon. In Yamada et al.’s [25] study, ultrasound examination was performed on each patient two days before or after colonoscopy. Not every patient was particularly prepared for the examination, but some of them received polyethylene glycol–electrolyte lavage. In Marin et al. [26], all the examinations were performed in a supine position after 15 min of rest and at least 6 h of fasting. 

#### 3.4.4. Sonoelastography Technique, Region of Interest, and Parameters of Sonoelastography

Before performing elastography, we conducted a US-B exploratory evaluation and searched for altered bowel segments.

Goertz et al. [24] utilised ARFI to measure the SWE. The Virtual Touch tissue quantification mode was used to perform ARFI measurements. During B-mode imaging, a 6.5 m ROI was centred within the intestinal walls, including the whole collapsed bowel section in a healthy bowel or mainly the anterior wall in a diseased bowel. During a relaxed pause in breathing, measurements were conducted in a longitudinal section of the dedicated bowel segment. At least 10 measurements were taken and recorded for each patient. The obtained measurements were then compared with the intramural wall thickness measurements obtained through standard ultrasound and the intramural semi-quantitative vascularisation according to Limberg et al. [29].

Rustemovic et al. [27] adopted the SE technique. Quantification of the elastography data was evaluated using the strain ratio (SR), which is the ratio of strain between two ROIs in the same image. To obtain EUS elastography SR, one ellipse was adjusted to the rectal wall, and a second one (same diameter) was adjusted to the surrounding tissue up to 15 mm from the rectal wall where the elastography signal was most obvious. The rectal wall tissue was used as the first ROI and the perirectal tissue as the second. The SR (rectal wall tissue strain %/perirectal tissue strain %) was calculated automatically.

Ishikawa et al. [28] also used the SE technique. The obtained images of the descending colon were classified into four types: normal, homogeneous, random, and hard. This classification was based on a colour scheme, in which researchers distinguished three types of colour systems: a homogeneous type, in which the thick wall was nearly completely green; a random type, in which the thick wall was imaged in various colours (red, green, or blue); and a hard type, in which the thick wall was nearly completely blue. Thus, green corresponded to normal, healthy, and non-thickened intestinal walls (< 4 mm), with a maintained five-layer structure. For the other types of images, the intestinal wall was thickened with non-preserved layering due to inflammation.

Yamada et al. [25] used the SWE technology in their study. B-mode images were used to delineate the bowel in the short axis, and the ROI measurement was set to a size large enough to contain the entire delineated bowel wall on the ventral side, including all layers from the mucosa to the serosa, avoiding the blood vessels. SWE/SWD measurements were performed using propagation displays and colour mapping, respectively. The measurements were performed at least five times. In the propagation view, if the ROI included a region with parallel contours of propagation with a constant interval, it was regarded as evaluable. Measurements were repeated a maximum of 10 times, and if a parallel region was acquired fewer than five times, the case was regarded as unevaluable. The intestinal wall thickness was measured manually as the perpendicular distance between the mucosa and the serosa on the screen on which the SWE/SWD was measured. 

Marin et al. [26] used ARFI to measure the SWE. For bowel elasticity, at least 10 measurements of the most affected bowel segment, also known as the ROI, were performed after IUS and Doppler ultrasound with a 4 C1 transducer; the average value was used. The ROI was placed at the position of maximum thickness, usually at 3 o’clock or 9 o’clock, incorporating as much bowel wall as possible without the surrounding tissues and luminal content. The results were expressed in metres per second.

## 4. Synthesis and Data Interpretation

Ishikawa et al. showed significant links between the elastography and colonoscopy results (*p* < 0.001). The colonoscopy results were classified into four groups: type A as healthy mucosa without erosion or ulceration, type B as mucosal oedema and erosion without ulceration, type C as punch-out ulcer, and type D as extensive ulceration. The results from the RTE examination were classified into four types: normal, homogeneous, random, and hard. Patients in the active phase of the disease presented abnormal elastographic results more often (*p* < 0.046) [28].

Rustemovic et al. showed a significant difference in the rectal wall thickness between patients with IBD, including UC, and patients from the control group without IBD (*p* = 0.001). Endoscopic examination of the patients with active inflammation in the course of UC in the rectum showed a much thicker rectal wall compared to the control group (median 4.5 mm vs. 3.6 mm; *p* = 0.03). No significant difference in the SR was detected. In the group of patients with UC, no significant difference was found in the rectal wall thickness and the deformation coefficient between patients with UC with active disease (*n* = 14) and patients with UC in remission (*n* = 1). The study also compared the thickness of the rectal wall and the strain coefficient in patients with CD and UC. A significant difference in deformation was observed between the group with active CD inflammation and the group with active UC inflammation (median 1.30 vs. 0.49; *p* = 0.0001). No significant difference in the rectal wall thickness was detected [27].

Significant differences were also found between patients with CD and UC in terms of age difference at diagnosis. The patients with CD were significantly younger at the moment of the final diagnosis than patients with UC (median 23.01 vs. 33.35 years; *p* = 0.016).

Goertz et al. showed no significant correlations between the ARFI values and body mass index, age, or intestinal thickness in any segment of the intestine. The ARFI values for specific parts of the intestine were as follows: terminal ileum (healthy 1.6 ± 0.35 (1.08–2.27) vs. UC 1.62 ± 0.53 (0.85–2.89), *p* = *n*.s.), ascending colon (1.96  ±  0.57 (1.36–3.29) vs. 2.10  ±  0.84 (1.29–4.12), *p* = *n*.s.), transverse colon (healthy 1.55  ±  0.37 (1.11–2.29) vs. 1.94  ±  0.60 (0.94–3.61), *p* = 0.045), descending colon (healthy 1.90  ±  0.71 (0.92–3.23) vs. 2.03  ±  0.71 (1.02–3.46), *p* = *n*.s.), and sigmoid colon (healthy 1.76  ±  0.55 (1.01–2.80) vs. 2.18  ±  0.51 (1.42–3.12), *p* = 0.032) [24].

A significant correlation was observed between the ARFI measurements of the sigmoid colon and the thickness of the sigmoid wall (*r* = 0.491; *p* = 0.028), but not with the Limberg scale. Neither the Mayo subscore nor the CRP levels were significantly correlated with any of the ARFI measurements of the intestinal segments. None of the other ARFI measurements correlated significantly with the wall thickness or the Limberg score in the corresponding segment of the intestine. ARFI elastography was not correlated with the depth of measurement in any of the bowel segments either in the healthy volunteers or in patients with UC. In the healthy group, the measurement depth of ARFI elastography in the descending colon (3.0 ± 0.8 cm) was significantly deeper than in the sigmoid colon (2.0 ± 0.7 cm) (*p* = 0.017), the ascending colon (*p* = 0.049), and the terminal ileum (*p* = 0.017). In the patient group, the measurement depth differed significantly (*p* < 0.001) only between the sigmoid (2.3 ± 0.7 cm) and the descending colon (3.1 ± 0.7 cm).

Yamada et al. used the Lichtiger index and the Ulcerative Colitis Endoscopic Severity Index (UCEIS) to assess the clinical severity of the disease. The Lichtiger index and UCEIS results were not correlated with the SWD values (*rs* = 0.004, *p* = 0.986 and *rs* = 0.002, *p* = 0.993, respectively). The Lichtiger index and the UCEIS results were moderately positively correlated (*rs* = 0.608, *p* = 0.001). There was no correlation between the wall thickness and the results of the SWE, SWD, Lichtiger index, or UCEIS (*rs* = −0.209, *p* = 0.306; *rs* = −0.010, *p* = 0.960; *rs* = 0.252, *p* = 0.214; and *rs* = 0.342, *p* = 0.087, respectively). The SWE values differed significantly between the two groups at 2.40 (IQR, 2.18–3.38) m/s and 1.62 (IQR, 1.44–1.95) m/s in the mucosa healing group and the active group (*p* = 0.007). There was no correlation between SWE and disease duration in the active phase group (*rs* = 0.080, *p* = 0.723) [25].

Marin et al. demonstrated a correlation between the ARFI and Mayo Scores in UC patients (*r* = 0.54, *p* = 0.0003), but also with intestinal wall thickness (*r* = 0.51, *p* < 0.0001) and faecal calprotectin (*r* = 0.40, *p* = 0.0006). They also identified the risk factors for active disease using multivariate logistic regression (odds ratio (OR) = 2.54, *p* = 0.0003), the Limberg score (OR = 3.80, *p* = 0.0003), bowel stiffness measured using ARFI elastography (OR = 21.75, *p* = 0.0012), and disease extension (OR = 2.61, *p* = 0.0022) [26].

## 5. Discussion

The diagnosis of UC is made on the basis of an overall interpretation of the clinical picture, laboratory tests, and endoscopic, histological, and radiological findings [30,31].

Currently, intestinal ultrasound training lacks well-defined standards, but steps are being taken to achieve consensus on what competencies should be expected from a certified IUS practitioner [32]. Standardised criteria for the ultrasound assessment of inflammatory activity in UC, such as the Humanitas Ultrasound Criteria or the Milan Ultrasound Criteria, have been established and verified [19,33]. Computer-assisted diagnosis systems have been designed to support less-experienced diagnosticians and reduce interobserver variability. We found several such endoscopy-based systems dedicated to UC [34,35,36], but to the best of our knowledge, there is no such intestinal ultrasound-based tool dedicated to this disease. Systems of this type dedicated to CD based on anal ultrasound have already been developed [37].

Despite the fact that research on UC and elastography is on the rise, with nearly 4000 and 2000 articles reported in 2021 by PubMed, respectively [38], the amount of works combining both is scarce. Thus far, systematic reviews on intestinal ultrasound have analysed both CD and UC and covered even fewer reports on the latter than our work [38]. The use of gastrointestinal ultrasonography in the diagnosis of IBD has been established; however, a reliable assessment of the usefulness of ultrasound elastography in the diagnosis and monitoring of UC seems impossible, and our observations are consistent with the current works [39,40,41]. However, based on the available reports, we can draw preliminary conclusions.

Currently, the specific section of the large intestine that should be the subject of the elastography examination has not been clearly determined, as with CD [42,43]. The distal colon seems to be the logical choice for an optimal evaluation localisation, but we do not know whether to evaluate the remodelled intestinal wall, the site of active inflammation, or both. Additionally, the diagnostic value of the transabdominal ultrasound in the rectal evaluation is reduced [44].

The transabdominal elastography examination did not differ significantly from the standard gastrointestinal ultrasonography examination in terms of the technique of performing and preparing the patient. During the examination, probes with a frequency above 7.5 MHz were used, allowing the assessment of the layers of the intestinal wall and the visualisation of focal lesions. Measurements were made in the longitudinal section of the intestine within its anterior wall, similar to elastographic studies in other regions [45,46]. The problem is the different depths of the position of the individual sections of the large intestine in transabdominal examination, which may significantly affect the measurements [17,47,48]. The lack of reports comparing various elastographic methods in the assessment of UC patients indicates a preferential diagnostic or monitoring technique is impossible. Most studies on intestinal elastography are based on the SE method due to its longer attainability and availability in endoscopic ultrasonography. The current guidelines recommend using the SE method to characterise bowel wall lesions in CD and point to insufficient evidence in relation to the SWE method [17].

Currently, assessing the correlation between the clinical scales used in the assessment of the disease and the elastographic results is difficult. However, current reports suggest that it is not possible to assess them solely on the basis of elastographic measurements.

Endoscopic ultrasonography can be used as an efficient modality to differentiate between CD and UC and to evaluate the disease activity [49]. At this point, it is unclear whether the extension of endoscopic ultrasound diagnostics with elastography can affect the accuracy of this assessment. Rustemovic et al.’s report gives reason to believe that such a distinction is possible based on elastography only, but further analysis is required. It should be noted that there are reports of increased sensitivity, specificity, and diagnostic accuracy with the simultaneous use of B-mode imaging, SE elastography, and contrast-enhanced ultrasound compared to the B-mode method alone in differentiating inflammatory from fibrotic ileal strictures among patients with CD [50]. This suggests the possibility of a similar relationship for UC.

According to the current guidelines, ultrasound has a limited role in the evaluation of intestinal UC complications [38]. The image of a toxic megacolon may show specific features, such as colonic dilatation (>6 mm) with thin walls (<2 mm) and fluid containment. Pseudopolyps or cancer shows irregular thickening of the wall or a pseudokidney sign [51,52]. During our analysis, we did not encounter any reports clearly supporting the possibility of assessing UC-specific complications with elastography. However, due to the proven value of elastography in the characterisation and staging of rectal tumours, there is a possibility that elastography could potentially be applicable in the detection or staging of cancer in the course of IBD in different localisations [17].

Finally, it is worth noting that, currently, apart from ultrasound elastography, magnetic resonance elastography is also a subject of research. This method offers the possibility of an elastographic evaluation of the intestines during magnetic resonance enterography. Reports show the possibility of a reliable IBD detection, the assessment of intestinal fibrosis in patients with CD, and worse outcome predictions. However, it is unable to reliably differentiate between UC and CD. Further research is required to accurately assess the clinical applicability of this method and its agreement with ultrasound elastography [53,54,55].

## 6. Study Limitations

This narrative review has several limitations. The main limitation is the small number of studies included in the review; therefore, the sample size is restricted. Due to the authors’ support for open access, this work did not include paid articles. Moreover, selected works differed significantly in terms of patient group, the type of elastography used, the measurement methodology, and the results’ presentation method. These reasons make it impossible to perform a comparative analysis between reports and a meta-analysis. Due to the use of various elastographic devices and measurement methods, the obtained measurements could not be reliably compared or the cut-off values could not be determined. The difference in the measurement results obtained with different elastographic techniques has already been proven. The lack of detailed information on ultrasound specialists performing the measurements made it impossible to assess operator-dependent errors in the analysed reports. The effect of disease duration and its severity on elastographic measurement remains unclear due to the lack of clearly presented data on this subject.

## 7. Conclusions

In conclusion, this narrative review provides an overview of the current (partial) evidence that abdominal elastography may be of diagnostic value in detecting fibrous bowel structures in UC patients. Elastography can potentially play an important role in the treatment of UC patients because it is a non-invasive, real-time, and inexpensive diagnostic technique and well tolerated by patients. It can help diagnose various fibrous and inflammatory structures of the bowels and, thus, help clinicians choose the best form of treatment. We believe that further research is necessary to clearly assess whether combining gastrointestinal ultrasonography with elastographic assessment can bring measurable benefits.

## Data Availability

A list of all analysed articles involved in the review is available from the authors.

## Supplementary Materials

The following supporting information can be downloaded at: https://www.mdpi.com/article/10.3390/diagnostics12092070/s1, Supplement S1: Checklist; Supplement S2: Search strategy.
