# Supplementary material for: Intestinal Elastography in the Diagnostics of Ulcerative Colitis: A Narrative Review
Source: Diagnostics (Basel). 2022 Aug 26;12(9):2070. doi: 10.3390/diagnostics12092070 (PMC9497506; doi:10.3390/diagnostics12092070)
Supplement: Supplementary file 1 [file diagnostics-12-02070-s001.zip › diagnostics-1812269-supplementary.pdf]

Checklist and Search strategy

General: Ultrasound Shear Wave OR elastography OR elastograms OR elastographies AND colitis ulcerative

Web of Science

First serch: 37,474 -> articles, 2010-2022, open access, radiology nuclear medicine medical imagining, English -> 567 *(after fitering)*

Colitis Ulcerative (Title) or Imaging Technique Elasticity (Title) or Tissue Elasticity Imaging (Title) or Elastography OR elastographic (Title) or Magnetic Resonance Elastography (Title) or Sonoelastography (Title) or Acoustic Radiation Force Impulse Imaging (Title) or ARFI Imaging (Title) or elastograms (Title) or ARFI (Title) or shear wave elastography (Title) or strain elastography (Title) or real-time elastography (Title) or Elasticity Imaging Technique (Title) and inflammatory bowel disease (Title) and Articles (Document Types) and Open Access and Radiology Nuclear Medicine Medical Imaging (Web of Science Categories) and English (Languages) and Open Access

Scopus

First serch: 555 -> open access, articles, 2010-2022, medicine, english -> 104 *(after fitering)*

(ALL (elasticity AND imaging AND technique) OR ALL (imaging AND technique AND elasticity) OR ALL (tissue AND elasticity AND imaging) OR ALL (elastography) OR ALL (elastographies) OR ALL (magnetic AND resonance AND elastography) OR ALL (sonoelastography) OR ALL (acoustic AND radiation AND force AND impulse AND imaging) OR ALL (arfi AND imaging) OR ALL (elastogram) OR ALL (arfi) OR ALL (shear AND wave AND elastography) OR ALL (strain AND elastography) OR ALL (real-time AND elastography) AND ALL (colitis AND ulcerative)) AND (LIMIT-TO (OA , "all")) AND (LIMIT-TO (PUBYEAR , 2021) OR LIMIT-TO (PUBYEAR , 2020) OR LIMIT-TO (PUBYEAR , 2019) OR LIMIT-TO (PUBYEAR , 2018) OR LIMIT-TO (PUBYEAR , 2017) OR LIMIT-TO (PUBYEAR , 2016) OR LIMIT-TO (PUBYEAR , 2015) OR LIMIT-TO (PUBYEAR , 2014) OR LIMIT-TO (PUBYEAR , 2013) OR LIMIT-TO (PUBYEAR , 2012)) AND (LIMIT-TO (DOCTYPE , "ar")) AND (LIMIT-TO (SUBJAREA , "MEDI")) AND (LIMIT-TO (LANGUAGE , "English"))

PubMed

First serch: 35 -> humans, 2010-2022, Abstract -> 17 *(after fitering)*

Elasticity Imaging Technique OR Imaging Technique Elasticity OR Tissue Elasticity Imaging OR Elastography OR Elastographies OR Magnetic Resonance Elastography OR Sonoelastography OR Acoustic Radiation Force Impulse Imaging OR ARFI Imaging OR ARFI Imagings OR Elastograms OR Elastogram OR ARFI OR shear wave elastography OR strain elastography OR real-time elastography AND Colitis Ulcerative

Cochranelibrary

First serch: 712 -> 2010-2022, trials -> 656 *(after fitering)*

Ultrasound Shear Wave OR elastography OR elastograms OR elastographies AND Colitis Ulcerative (search with variations turned on)
